# Diagnosis and Management of Inborn Errors of Metabolism in Adult Patients in the Emergency Department

**DOI:** 10.3390/diagnostics11112148

**Published:** 2021-11-19

**Authors:** Isabel Solares, Carlos Heredia-Mena, Francisco Javier Castelbón, Daniel Jericó, Karol Marcela Córdoba, Antonio Fontanellas, Rafael Enríquez de Salamanca, Montserrat Morales-Conejo

**Affiliations:** 1 Department of Internal Medicine, University Hospital, 12 de Octubre, Av. de Córdoba, s/n, 28041 Madrid, Spain; isolares@alumni.unav.es (I.S.); cherediam@salud.madrid.org (C.H.-M.); fco.javier.castelbon@salud.madrid.org (F.J.C.); salamanca@med.ucm.es (R.E.d.S.); 2 National Reference Center for Congenital Errors of Metabolism (CSUR) and European Reference Center for Inherited Metabolic Disease (MetabERN), University Hospital, 12 de Octubre, 28041 Madrid, Spain; 3 Hepatology Program, Centre for Applied Medical Research (CIMA), University of Navarra, 31008 Pamplona, Spain; djerico@alumni.unav.es (D.J.); kcordoba@alumni.unav.es (K.M.C.); afontanellas@unav.es (A.F.); 4 Instituto de Investigación Sanitaria de Navarra (IdiSNA), 31008 Pamplona, Spain; 5 Centro de Investigación Biomédica en Red de Enfermedades Hepáticas y Digestivas (CIBEREhd), Instituto de Salud Carlos III, 28029 Madrid, Spain; 6 Spanish Network for Biomedical Research in Rare Diseases (CIBERER), U723, Instituto de salud Carlos III, 28029 Madrid, Spain; 7 Research Institute “i+12”, University Hospital, 12 de Octubre, 28041 Madrid, Spain

**Keywords:** inborn errors of metabolism, acute porphyrias, mitochondrial disease, urea cycle disorders

## Abstract

Inborn errors of metabolism (IEM) constitute an important group of conditions characterized by an altered metabolic pathway. There are numerous guidelines for the diagnosis and management of IEMs in the pediatric population but not for adults. Given the increasing frequency of this group of conditions in adulthood, other clinicians in addition to pediatricians should be aware of them and learn to identify their characteristic manifestations. Early recognition and implementation of an appropriate therapeutic approach would improve the clinical outcome of many of these patients. This review presents when and how to investigate a metabolic disorder with the aim of encouraging physicians not to overlook a treatable disorder.

## 1. Introduction

Inborn errors of metabolism (IEM) encompass a group of genetic diseases characterized by a partial deficit or altered structure which affect the function of a protein and cause the blockage of a metabolic pathway. Interference in these enzyme pathways results in the deficiency of a particular end product or the excessive accumulation of an intermediate substrate, which can be toxic [1]. Most of the IEMs are transmitted as autosomal-recessive traits.

The importance of providing health professionals who care for adult patients with basic knowledge of IEMs is based on three principles; despite their low individual prevalence, together they can affect up to 1/800 live births [2]. Furthermore, the prognosis of this type of disease has been improving over the years due to the implementation of neonatal screening that allows some subtypes to be detected early, in addition to advances in their diagnosis and treatment. Currently, there is an increasing number of patients with IEMs who reach adulthood [3]. However, some IEMs can begin in adulthood, either with slowly progressive onset disorders, such as lysosomal diseases, or with episodes of acute decompensation, characteristic of acute porphyria attacks or urea cycle disorders [4]. 

The diagnosis of an IEM can be challenging due to their nonspecific clinical presentation and the lack of experience of physicians with this type of disorder [5]. Although these diseases have a wide clinical spectrum, the nervous and gastrointestinal systems are those most frequently observed in IEMs. Neurological involvement can manifest both as progressive degenerative symptoms (developmental delay, movement disorders, hypotonia, etc.) and acute episodes of encephalopathy (coma, seizures, psychiatric symptoms, etc.) [6,7]. Gastrointestinal involvement is also common and includes recurrent episodes of vomiting or severe abdominal pain, aversion to protein foods or hepatomegaly, among others. 

**Figure 1:**
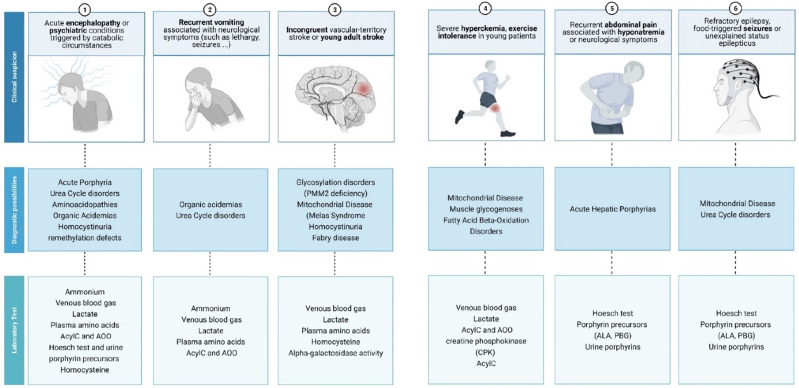
How to address a suspected inborn error of metabolism error in the emergency department.

Prompt and effective care is essential in patients with an IEM since therapeutic delay has been associated with the development of fatal events that include severe metabolic acidosis, hyperammonemia, cerebral edema and death [8]. Therefore, in the emergency department, it is crucial to be aware of these entities, to provide early and effective treatment during acute decompensations in patients with a known IEM and to suspect a possible IEMs in those patients without an established diagnosis [9] (Figure 1).

## 2. Nosological Classification

**Figure 2:**
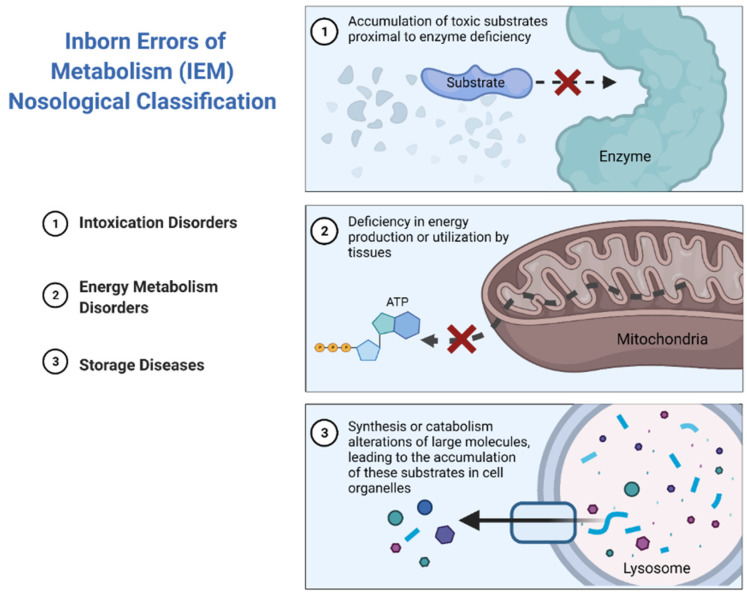
Inborn errors of metabolism nosological classification.

The major subtypes of IEMs can be classified into three broad groups according to similarities in pathogenesis and/or presenting features [10] (Figure 2).

**Figure 3:**
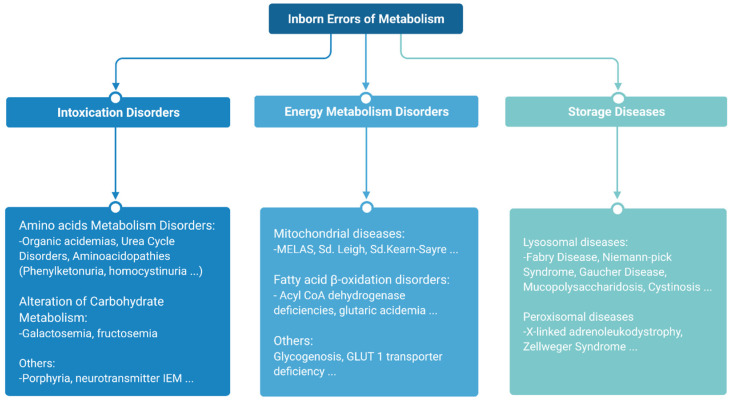
IEM classification according to symptoms.

1. Intoxication disorders: The accumulation of toxic compounds proximal to the metabolic blockage is responsible for the symptoms in these disorders. Usually, patients alternate between symptom-free periods and acute decompensations triggered by the factors specific to each disease [11].
2. Energy metabolism disorders: Disorders caused by a disruption of either production or utilization of energy by tissues fall in this category. This compromises those organs with the highest energy consumption such as the heart, skeletal muscle or the brain [12,13].
3. Storage diseases: These disorders are caused by alterations in the synthesis or catabolism of large molecules, which lead to the accumulation of these substrates in cell organelles, resulting in lysosomal or peroxisomal malfunction, which ultimately results in cell dysfunction and cell death [14]. This subgroup rarely leads to acute decompensations requiring urgent management, with the exception of strokes in patients with Fabry disease [15], or acute adrenal insufficiency in the case of X-linked adrenoleukodystrophy [16] (Figure 3).

## 3. General Approach 

Patients with an IEM who come to the emergency service for a possible decompensation require urgent assessment and treatment, since the patient’s life can be compromised in a few hours due to the rapidity of the decompensation in this type of disorder. These patients often bring protocols made specifically for them in their reference metabolic units [17]. Otherwise, there are metabolic guides available online that can provide useful recommendations for the management of these disorders [18].

The first signs of decompensation can be subtle, so the clinician should be alert to symptoms such as lethargy, loss of appetite, change in behavior or exacerbation of pre-existing neurological problems (irritability, movement disorders, seizures, etc.). Those circumstances that prevent adequate feeding of patients, such as vomiting or drowsiness, should always be taken seriously.

The biochemical alterations that usually accompany this type of entity include hyperammonemia, lactic acidosis, hypoglycemia, or rhabdomyolysis [19,20]. Note that, at an early stage, plasma ammonia concentration levels are not a reliable biomarker. Neurological symptoms are likely due to brain glutamine accumulation, which precedes plasma ammonia increases. Therefore, we must be guided by symptoms rather than analytical parameters when grading the severity of this condition.

Early signs of decompensation require immediate action. We recommend the following general approach:

(A) Given the frequent association of certain triggering factors with the development of acute decompensations, it is essential to inquire about the possible implication of catabolic situations (infections, fasting, physical activity, childbirth, etc.), the intake of food that cannot be metabolized by the patient (proteins, fatty acids, etc.), or certain drugs, when facing an acute crisis in patients with an IEM [21]. If possible, these factors should be quickly removed when possible.
(B) Laboratory evaluation: Blood analyses that include ammonia, blood gas, lactate, hemogram and biochemistry, as well as determination of ketone bodies in the urine, should be obtained. When an IEM is suspected, although it is not usually possible to make a confirmatory genetic diagnosis in the emergency department, it is advisable to send 3 mL of blood in an EDTA tube, urine and blood on filter paper to a laboratory specialized in IEMs. If this cannot be sent immediately, 3 mL of blood should be collected in an EDTA tube; then, this should be centrifuged, the plasma separated and the whole sample frozen together with a urine specimen.
(C) Catabolism should be avoided by providing 10% dextrose at a rate of 3–4 mg/kg/min starting as soon as possible. Due to its ability to aggravate hyponatremia by dilution, 10% dextrose should be administered with caution. This is especially relevant in acute intermittent porphyria (AIP; MIM: 176000), where hyponatremia plays a central role in patient prognosis.
(D) Sodium inputs can be administered with additional 0.9% NaCl or via sodium chloride ampoules added to 10% dextrose to avoid adding excess volume to the patient.
(E) If blood glucose consistently exceeds 200 mg/dL, it is advisable to prioritize insulin treatment rather than reducing glucose intake. Treatment with insulin should be started at doses of 0.01–0.02 IU/kg/h to maintain blood glucose levels between 120 and 200 mg/dL.
(F) Hyperammonemia and lactic acidosis should be urgently corrected. Metabolic acidosis can be treated with bicarbonate. In the case of hyperammonemia lower than 80 μmol/L, the restriction of dietary protein intake should be sufficient. When levels are between 80 and 200 μmol/L, the use of ammonium chelators is advisable (see specific information in this regard or consult the metabolic unit). In the case of impaired consciousness or hyperammonemia higher than 200 μmol/L, hemofiltration should be considered.
(G) Treatment with specific cofactors and detoxifiers according to the particular disease in question should be carried out.

## 4. Intoxication Disorders

Sometimes, this subset of IEMs manifests as acute metabolic emergencies which can lead to permanent sequelae and even death, if undetected and inappropriately treated by the physician.

The most common laboratory findings of these decompensations are lactic acidosis and hyperammonemia [19]. 

Pyruvate is produced in the cytoplasm from glucose during glycolysis. Then, pyruvate enters the mitochondria, where it is converted to acetyl-CoA by the pyruvate dehydrogenase complex. Acetyl-CoA enters into the tricarboxylic acid cycle (TCA) and, ultimately, provides metabolites to the respiratory chain where the majority of energy is produced. Pyruvate may also be converted back to glucose by gluconeogenesis. When the normal metabolic path of pyruvate is blocked, it may be converted into lactic acid [22].

IEMs which can lead to hyperlactacidemia are those that cause dysfunction in any of the four primary biochemical pathways linked to lactate metabolism: glycolysis (which catalyzes the conversion of glucose to pyruvate and thence to lactate), the TCA (that transforms acetyl-CoA to CO2 and the reducing agents NADH and FADH), the mitochondrial electron transport chain (transferring electrons donated by NADH and FADH2 to O2, producing ATP) and gluconeogenesis (where lactate is converted back to glucose via pyruvate in the liver) [23]. Sodium bicarbonate is not routinely given, as acidosis usually corrects rapidly after infusion of intravenous (i.v) fluid therapy, but sodium bicarbonate may be needed if the pH is lower than 7.1, in cases where the pH is rapidly deteriorating, or the baseline deficit is greater than 15 mmol/L.

Acute porphyrias could be also considered intoxication disorders. However, due to their peculiar diagnostic–therapeutic approach, they are specifically dealt with in a separate section.

### 4.1. Specific Approach to Hyperammonemia in Intoxication IEM Disorders

Ammonia is a neurotoxic compound which is detoxified through the urea cycle. Hyperammonemia is a potentially life-threatening complication mainly observed in the congenital defects of the urea cycle, classical organic acidurias and the defects of mitochondrial fatty acid oxidation. Duration and severity of hyperammonemia strongly correlates with brain damage, so effective and prompt diagnosis and treatment is crucial to prevent irreversible neurological damage [24].

If hyperammonemia is confirmed, the determination of plasma amino acids, acylcarnitine, urinary organic acids and orotic acid should be urgently requested together with basic laboratory investigations. 

The emergency management of hyperammonemia in IEMs is based on three interdependent principles: the reversal of the catabolic state through caloric supplementation; the pharmacological uptake of excess nitrogen species; and the physical removal of the ammonia by dialysis or hemofiltration, if needed [25]. 

Accordingly, it is essential to reduce both endogenous and exogenous ammonium contributions. Proteins should be removed from the diet for a period of 24–48 h and fluid therapy with 10% Dextrose (2 cc/kg/hour) should be provided, to avoid protein catabolism. Pharmacological treatment is based on either ammonium derivation into non-toxic products or optimization of the urea cycle to promote its metabolism.

**Figure 4:**
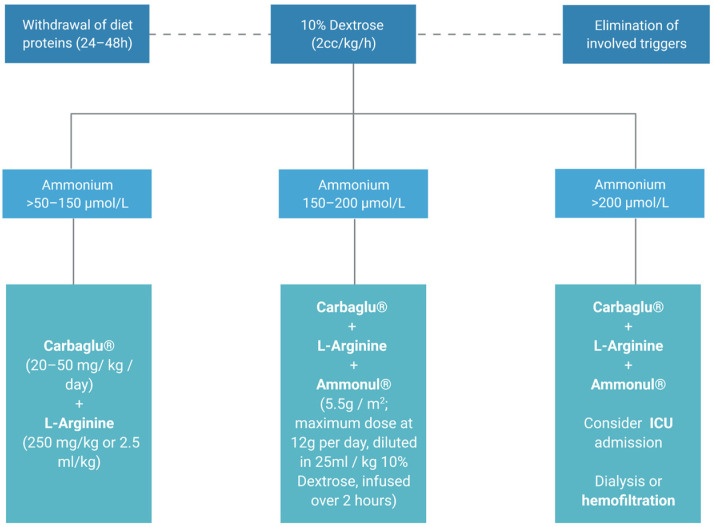
Recommendation of an hyperammonemia approach in the emergency department.

When hyperammonemia is higher than 50–150 µmol/L, administration of the cofactor N-carbamylglutamate (Carbaglu^®^, Recordati, Italy; 100 mg/kg oral 1st dose, 100–250 mg/kg/day orally in 2–4 doses, maintenance until normalization) is recommended together with l-Arginine (700 mg/kg/day). In the case of hyperammonemia associated with urea cycle disorders, Carbaglu is not effective and chelators should be used. For ammonia elimination in hyperammonemia levels higher than 150 µmol/L, in addition to the previously described measures, sodium benzoate and sodium phenylacetate can be combined (Ammonul^®^, Ucyclyd Pharma, Inc., Scottsdale, AZ, USA; 5.5 g/m^2^ maximum dose 12 g diluted in 25 mL/kg of SG 10% to pass 2 h), which must be administered i.v through a central venous line. Given the susceptibility of adults to developing intracranial hypertension and cerebral edema with hyperammonemia, dialysis should be started if ammonia exceeds 200 μmol/L (Figure 4) [26]. In all cases of hyperammonemia, the administration of Valproate, Midazolam, Acetylsalicylic acid and Pivampicillin is prohibited [27]. 

### 4.2. Approach to Acute Porphyria Attacks

Porphyrias are inborn errors in the heme metabolic pathway. Symptoms are determined by the accumulated intermediate products. Acute porphyrias (AIP), hereditary coproporphyria (HC), variegate porphyria (VP) and δ-aminolevulinic acid (ALA) dehydratase deficient porphyria (ADP) manifest as acute neurovisceral crises secondary to the accumulation of the porphyrin precursors, ALA or both ALA and porphobilinogen.

Cutaneous porphyrias are characterized by the overproduction of porphyrins, whose skin deposition causes dermal lesions due to their characteristic photosensitivity. HC and VP are called mixed porphyrias due to their ability to produce both symptoms. AIP is the most common subtype of acute porphyria and causes the most severe neurovisceral attacks [28]. Of note, a high percentage of acute attacks are triggered by porphyrinogenic factors such as fasting, alcohol consumption, infections, hormonal factors or the consumption of certain drugs. Identifying and eliminating potential triggers is indispensable. 

Acute porphyria attacks are characterized by neurovisceral crises involving the autonomic, peripheral and central nervous systems. They manifest as severe abdominal pain accompanied by nausea, occasional vomiting and constipation, peripheral neuropathy and psychiatric disorders (such as anxiety and insomnia, agitation and/or hallucinations). Tachycardia, tendency to hypertension and hyponatremia are common. Severe hyponatremia represents a marker of severity of the porphyria attack and must be carefully managed, since it favors the development of seizures [29]. A therapeutic option in acute porphyria attacks complicated by the development of hyponatremia lower than 130 mEq/L is the use of Tolvaptan^®^ [30].

If the porphyric attack is not aborted quickly and adequately, it can progress to complete tetraparesis and respiratory muscle dysfunction.

**Figure 5:**
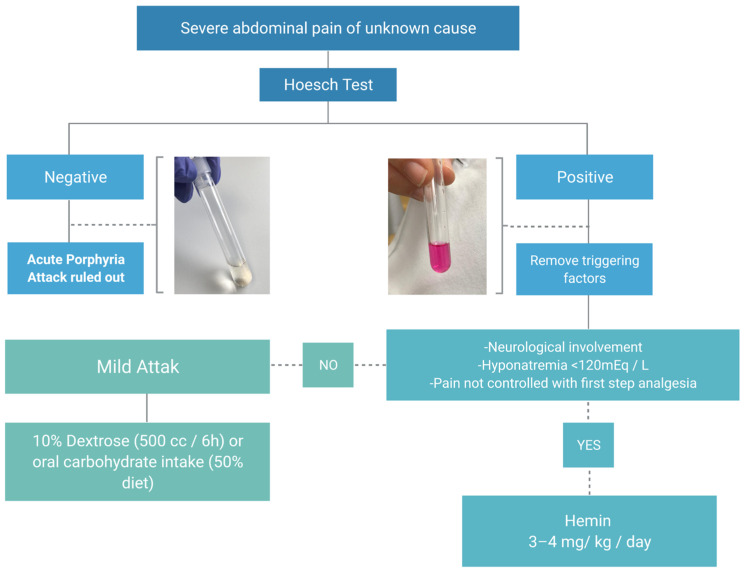
Recommended approach for the acute porphyria attack in the emergency department.

The characteristic laboratory findings during the attack are a marked increase in plasma and urinary concentrations of ALA and PBG. Urinary PBG excess can be easily identified by the Hoesch test (after adding two drops of urine to 1 mL of Ehrlich reagent, the reagent immediately stains pinkish red at high concentrations of urinary PBG). A negative test rules out that the abdominal pain is due to a current porphyric crisis. Treatment of a mild crisis of acute porphyria is based on the i.v administration of carbohydrates, whereas, for moderate and severe attacks, the recommendation is to use hemin (Normosang^®^, Recordati, Italy) [31] (Figure 5).

## 5. Energy Metabolism Disorders

This group of IEMs has a very broad clinical and genetic spectrum, but an alteration in ATP production underlies all of them. It includes fatty acid Beta-oxidation disorders, as well as mitochondrial and glycogen storage diseases. Symptoms may vary, but cardiac, musculoskeletal and nervous systems are those mainly affected. HyperCKemia, mild hyperammonemia, hypoglycemia and hyperlactacidemia are common biochemical features in these diseases. Energy disorders that may require urgent management are briefly discussed.

### 5.1. Fatty Acid Beta-Oxidation Disorders

Fatty acids are essential nutrients whose storage as triglycerides in adipose tissue allows humans to tolerate periods of low circulating glucose levels, such as prolonged fasting episodes, and other energy-intensive conditions, such as fever or exercise. The main route of degradation of long-chain fatty acids is the β-oxidation of mitochondrial fatty acids (FAO), which, in addition to fueling the TCA and oxidative phosphorylation, also stimulates the hepatic synthesis of ketone bodies [32].

Defects in this metabolic pathway promote the dysfunction of gluconeogenesis, ammonium metabolism and the formation of ketone bodies, which leads to episodes of non-ketonic hypoglycemia, hyperammonemia and lactic acidosis in catabolic scenarios [33]. Liver, heart and muscle are the most dependent organs on the fatty acid beta-oxidation pathway for energy production, so it is common in patients with this IEM to present hepatomegaly and/or acute liver failure, cardiomyopathy and rhabdomyolysis [34]. Long-term treatment consists of avoiding prolonged fasting and dietary restriction of non-metabolizable triglycerides. Many patients with a defect in one of the long-chain-specific enzymes have medium-chain triglycerides which supply a fatty acid substrate that can bypass the enzymatic defect [35]. Carnitine supplementation is crucial in patients with primary carnitine deficiency, but there is no evidence of benefit in other fatty acid disorder subtypes.

During acute decompensation, fat intake should be interrupted and a sufficient amount of oral or i.v. glucose should be administered to avoid lipolysis and to maintain an adequate state of hydration, given the risk of rhabdomyolysis in these patients. Carnitine treatment should be maintained in those patients who receive it regularly and, in the case of pH lower than 7.20, the i.v. administration of sodium bicarbonate is recommended. Although hyperammonemia is usually mild and resolves with standard measures, in the case of moderate–severe hyperammonemia, adding carbamylglutamate should be considered [26].

### 5.2. Mitochondrial Disease

Mitochondrial disease encompasses a heterogeneous group of genetic disorders characterized by defective oxidative phosphorylation, resulting in inefficient energy production and alteration in the reduction–oxidation state. Although, individually, they have a low prevalence, as a whole, it is the most prevalent subgroup within inborn errors of metabolism. In fact, it is estimated that mtDNA mutation frequency in the healthy population is approximately 1 in 200, although many of these cases have no clinical expression of the disease due to their low percentage of heteroplasmy [36].

Mitochondrial diseases display a wide clinical heterogeneity due to the high copy number of mtDNA in human cells, which can consequently contain both mutant and wild-type mtDNA populations. Although individuals might present with a constellation of clinical features, the most common presentations in adults are myopathies, progressive encephalopathies or ataxias and stroke-like episodes. In addition, short stature, sensorineural deafness, diabetes and pigmentary retinopathy frequently accompany the multisystem clinical spectrum of these patients. 

Among long-term management measures, it is essential to avoid triggering factors (such as drugs which inhibit the mitochondrial respiratory chain, e.g., valproic acid, carbamazepine, aminoglycosides, tetracycline or ciprofloxacin, propofol, etc.). Dietary supplements to optimize the function of the mitochondrial respiratory chain should be added (Coenzyme Q, thiamine, niacin, etc.), or agents that reduce the accumulation of toxic metabolites (l-carnitine) should be provided [37].

The mitochondrial encephalomyopathy, lactic acidosis and stroke-like episodes (MELAS) syndrome is characterized by mitochondrial myopathy, encephalopathy, lactic acidosis and the development of acute stroke-like episodes, which typically do not correspond to a certain vascular territory [38]. In this scenario, the intravenous administration of l-Arginine 10% (10 g/m^2^, diluted in 5% Dextrose infused in 2 h and subsequently a maintenance dose of 10 g/m^2^/day for 1–3 days) is recommended.

## 6. Pregnancy in a Patient with an IEM

As more patients with an IEM are reaching childbearing age, the clinical and therapeutic challenges in these patients have increased for clinicians. Women with an IEM should always plan their pregnancies and be closely monitored. Dietary management is complex and requires a multidisciplinary approach by experienced specialists; however, certain considerations should be known by all those doctors who may treat these patients in the emergency service [39]. 

Patients with homocystinuria have an increased risk for thrombus formation. Given that pregnancy is a procoagulant situation, a possible thrombotic complication should be suspected in these patients under certain suggestive clinical scenarios (cavernous sinus thrombosis in patients with high intensity headache, pulmonary thromboembolism in acute respiratory failure, etc.) [40,41,42].

Likewise, we must rule out the presence of a urea cycle disorder (specifically, a deficit of Ornithine TransCarbamylase, MIM: 300461) in those patients who present symptoms of drowsiness, or behavioral alteration during pregnancy or delivery, by checking for the presence of hyperammonemia.

Pregnancy can be a dangerous period for women with a urea cycle disorder. Nausea and vomiting can reduce energy intake, therefore promoting episodes of metabolic decompensation. It is essential to avoid dehydration in these patients and, in the case of severe decompensation episodes, immediate measures should be adopted to avoid catabolism by administering i.v. dextrose therapy. In the case of hyperammonemia, l-arginine/l-citrulline and sodium benzoate therapy are safe drugs during pregnancy [43].

On the other hand, it is important to consider an as-yet-undiagnosed IEM in all pregnant women with acute liver failure, preeclampsia, or HELLP syndrome (the acronym for hemolysis elevated liver enzymes and low platelet count) during the third trimester. The possibility of an underlying β-oxidation disorder, especially long-chain 3-hydroxy acyl CoA dehydrogenase (LCHAD) deficiency, should be ruled out, due to the homozygous fetus accumulating long-chain 3-OH-acyl, which, added to the obligate carrier condition of the mother, induces a hepatotoxic effect on her [44].

## 7. Conclusions

In view of major improvements in the diagnosis and treatment of IEMs that have led to an increasing prevalence of affected patients reaching adulthood, it has become crucial for clinicians to recognize these diseases in adults.

In this review, we summarize the main acute presentations of IEMs and offer recommendations for their diagnosis and treatment in the emergency department, in order to prevent a treatable disorder from being overlooked.
